# Effects of empagliflozin and metformin on biofilm formation and pathogenicity factors of urinary *Escherichia coli* isolates

**DOI:** 10.1007/s12223-026-01520-3

**Published:** 2026-06-03

**Authors:** Aybala Temel, Ayşegül Ateş, Zinnet Şevval Aksoyalp

**Affiliations:** 1 Department of Pharmaceutical Microbiology, Faculty of Pharmacy, Izmir Katip Çelebi University, 35620 Izmir, Türkiye; 2 Department of Pharmaceutical Microbiology, Faculty of Pharmacy, Ege University, 35010 Izmir, Türkiye; 3 Department of Pharmacology, Faculty of Pharmacy, Izmir Katip Çelebi University, 35620 Izmir, Türkiye

**Keywords:** Antibiofilm, Antidiabetics, Antimicrobial, Antivirulence, Drug repurposing, Empagliflozin, Metformin, Non-antibiotic drugs, SGLT-2 inhibitors

## Abstract

*Escherichia coli*, a major cause of urinary tract infections (UTIs), forms biofilms that contribute to antimicrobial resistance. Antidiabetic medications have gained attention for their potential antimicrobial effects, though data remain limited. This study investigated the inhibitory effects of empagliflozin and metformin against urinary *E. coli* isolates. Minimum inhibitory concentrations (MICs) were determined via broth microdilution, and synergistic interactions were assessed using the checkerboard method. Biofilm inhibition at sub-inhibitory drug concentrations was evaluated spectrophotometrically, and gene expression of *fimH* and *luxS*, were analyzed using RT-qPCR. Empagliflozin and metformin inhibited bacterial growth, with MICs ranging from 3.12–6.25 mg/mL and 25–50 mg/mL, respectively. A synergistic effect was observed in two isolates. Both drugs significantly reduced biofilm formation (51.8–72.9%) and downregulated *fimH* and *luxS* gene expression (*p* < 0.01). This study showed that empagliflozin and metformin could have inhibitory effects against urinary *E. coli* isolates*,* supporting their potential in drug repurposing strategies. Empagliflozin and metformin demonstrated significant dose-dependent in vitro antivirulence and antibiofilm activities, further supported by the downregulation of key virulence-associated genes (*fimH* and *luxS*). To the best of our knowledge, this is the first report investigating the in vitro effects of empagliflozin against urinary *E. coli* isolates, and further investigation is required to determine the impact of antidiabetic medications on *E. coli.*

**Supplementary Information:**

The online version contains supplementary material available at https://doi.org/10.1007/s12223-026-01520-3.

## Introduction

Antimicrobial resistance (AMR) leading to a decrease in effective treatment agents requires urgent priority to identify new chemical compounds to combat infections**.** In recent years, concerns have been raised over a decline in the number of approved antibiotics, concomitant with an increase in the number of multidrug-resistant bacteria (Serafin and Horner 2018; Anwer and Ahmed 2024). Urinary tract infections (UTIs) are among the most common bacterial infections, with uropathogenic *Escherichia coli* (UPEC) responsible for the majority of cases. UPEC expresses multiple virulence factors—such as adhesins, toxins, and biofilm-forming capability—that promote colonisation, persistence, and immune evasion (Mlugu et al. 2023). Biofilm formation not only enhances bacterial survival in the urinary tract but also limits antibiotic penetration, contributing to recurrent infections and AMR. Increasing resistance rates among UPEC isolates underscore the need for new antimicrobial strategies. The classical drug development process involves numerous preclinical and clinical studies and requires a long time and substantial investment of financial resources (Langedijk et al. 2015). Drug repurposing strategy offers a practical approach by utilising the known pharmacological and safety profiles of approved drugs, thereby bypassing the lengthy and costly traditional drug development pathway (Oprea et al. 2011; Kepplinger 2015). In the face of the global AMR crisis, exploring the antimicrobial properties of non-antibiotic drugs offers a promising and cost-effective strategy (Oprea et al. 2011; Kepplinger 2015; Langedijk et al. 2015).

Several drugs for the treatment of noninsulin-dependent diabetes mellitus, including metformin, have demonstrated antimicrobial or immunomodulatory potential, supporting their repurposing (Hegazy et al. 2021; Tarin-Pello et al. 2025). Sodium/glucose cotransporter-2 (SGLT-2) inhibitors lower blood glucose by inducing glycosuria, but their association with UTIs remains uncertain, with studies reporting inconsistent findings (Zaccardi et al. 2016; Li et al. 2017; Liu et al. 2017; Uitrakul et al. 2022; Kittipibul et al. 2024). Evidence regarding the antimicrobial or antibiofilm properties of SGLT-2 inhibitors is limited and predominantly centred on canagliflozin (Gu et al. 2023). Moreover, SGLT-2 inhibitors are commonly prescribed alongside metformin, and recent adverse event analyses suggest that this combination may be associated with a lower frequency of UTIs (Liu et al. 2017), although their combined antimicrobial effects remain unclear. Recent findings indicate that the association between SGLT-2 inhibition and infection risk is more complex than initially hypothesised; this underscores the necessity for further exploration into the direct interactions between these antidiabetic medications and uropathogens at the molecular level (Zaccardi et al. 2016; Li et al. 2017; Liu et al. 2017; Kittipibul et al. 2024). This preliminary investigation aimed to evaluate in vitro antibacterial and antibiofilm activity of empagliflozin and metformin against urinary *E. coli* isolates and their virulence-associated genes.

## Materials and methods

### Bacterial strains and drugs

Forty *E. coli* strains isolated from clinical urine specimens at the Bacteriology Laboratory of the Department of Medical Microbiology, Faculty of Medicine, Ege University, Izmir, Türkiye were included in this study (Supplement Fig. 1). *E. coli ATCC 25922, Enterococcus faecalis ATCC 29212*, and *Pseudomonas aeruginosa ATCC 27853* were internal quality control strains. Identification and antimicrobial susceptibility profiles of clinical *E. coli* isolates were detected by using VITEK-2 (AST-N326 and AST-N327 cards; bioMérieux, France) and BD Phoenix™ M50 ID/AST (NMIC/ID-204 panels; Becton Dickinson, Maryland, USA) automated systems (Supplement Table 1). *E. coli* isolates were stored at −80 °C in brain–heart infusion broth (with 10% glycerine). The significant growth thresholds for *E. coli* isolates, as defined by the NHS (National Health Service, UK) and UK Standards for Microbiology Investigations (UK SMI), were utilized to verify their status as urinary strains in this study (Supplement Table 1). According to these guidelines and interpreting criteria, clinical *E. coli* isolates with growth threshold values of ≥ 10^3^ CFU/mL were selected for inclusion in this study. Polymerase Chain Reaction (PCR) was performed to identify various virulence genes (*hly, cnf,* and *pap*) in five representative urinary *E. coli* strains for confirmation (Supplement Fig. 2).

**Fig. 1:**
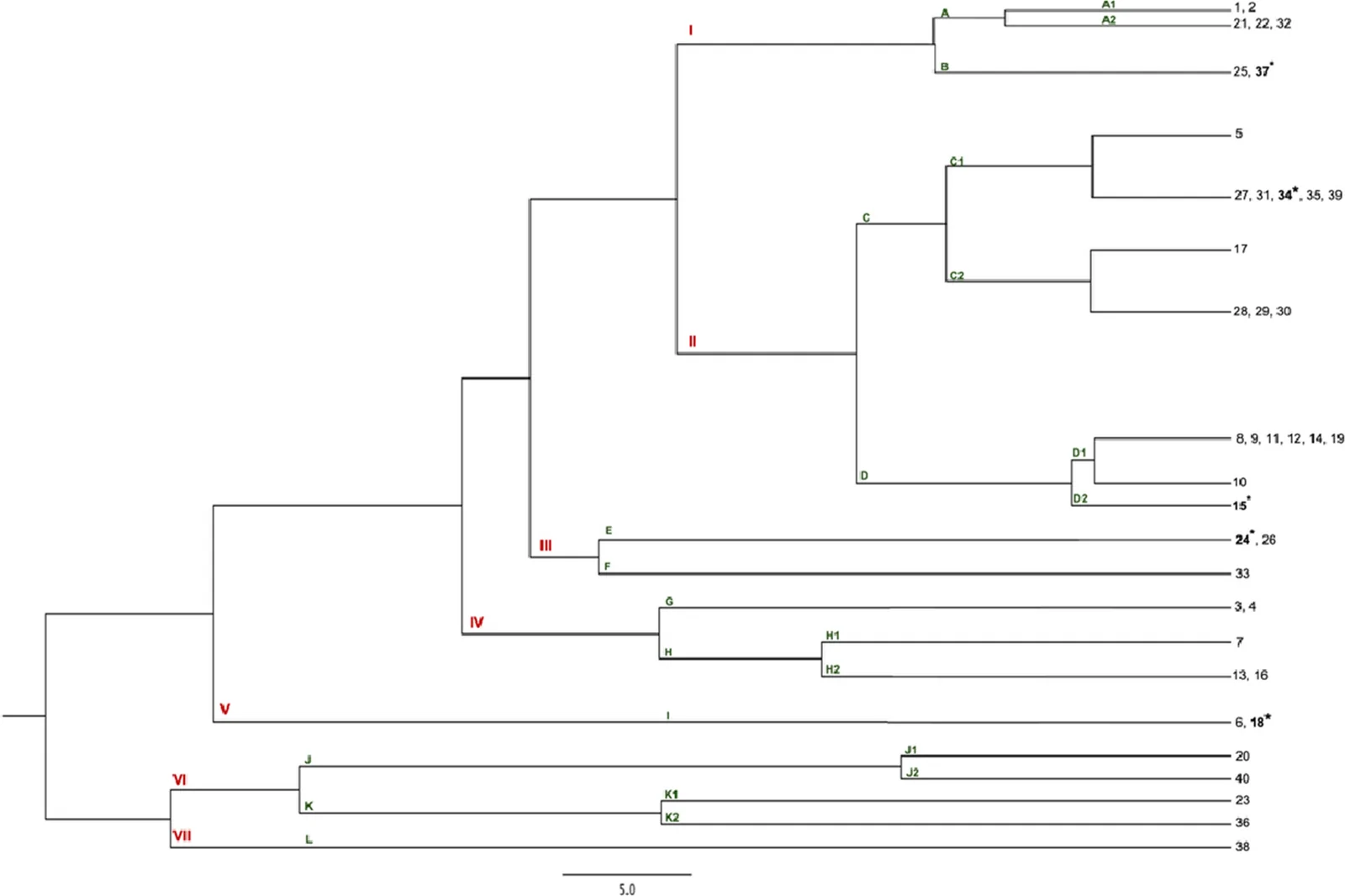
ERIC-PCR dendrogram of clonal relationships of clinical *E. coli* isolates (I-VII: main groups, A-L: clones)

After the initial confirmation of the isolates, all 40 isolates were subjected to Enterobacterial Repetitive Intergenic Consensus (ERIC)-PCR, which identified 12 distinct clonal groups. To ensure representative genetic diversity for subsequent experiments, two isolates were selected from each of the 12 clones (n = 24) to undergo biofilm formation assays. Following phenotypic characterization, five isolates (E15, E18, E24, E34, and E37) exhibiting robust biofilm-forming capacities were strategically selected from different genetic clones for comprehensive analysis, including Minimum Inhibitory Concentration (MIC) determination, checkerboard synergy assays, and gene expression studies. To ensure a comprehensive and unbiased representation of the isolate population, these five isolates were chosen based on three main criteria: (i) phenotypic diversity in biofilm production (ranging from moderate to strong), (ii) distinct genetic backgrounds as confirmed by ERIC-PCR clonal analysis (representing different clones such as B, C, D, E, and I; see Fig. 1), and (iii) varied antibiotic susceptibility profiles, including multi-drug resistant (MDR) strains (Supplement Fig. 1). This selection strategy was implemented to eliminate selection bias and to ensure that the findings reflect the broad genetic and phenotypic spectrum of clinical isolates. To ensure the accuracy of the resistance profiles and address the inherent limitations of automated systems, the antibiotic susceptibilities of the five representative isolates (E15, E18, E24, E34, and E37) were further validated using the reference broth microdilution and disk diffusion methods in accordance with EUCAST guidelines.

Empagliflozin (EMP) (MedChemExpress, USA) and metformin (MET) (Abdi Ibrahim Pharmaceuticals, Türkiye) were commercially available at appropriate analytical purity. Agent solutions were prepared fresh in accordance with the recommendations of manufacturers in ultrapure water and/or DMSO (< 15% of the final volume). DMSO control groups were included in the antimicrobial and antibiofilm activity experiment for each run. For the gene expression analysis, all transcriptional data were normalized to the DMSO control groups (Supplement Table 5). Ciprofloxacin (CIP, Fisher Scientific, USA) was used for antibiotic susceptibility and checkerboard assays. Potency calculations were performed for antibiotic solutions, and all agent solutions were filtered to the appropriate pore size before use. The stock solutions were stored at −20 °C.

### Determination of clonality by ERIC-PCR

Clonal relationships of clinical *E. coli* isolates (n = 40) were determined by Enterobacterial Repetitive Intergenic Consensus Polymerase Chain Reaction (ERIC-PCR) using the primers 5′-ATGTAAGCTCCTGGGGATTCAC-3′ ERIC 1 and 5′-AAGTAAGTGACTGGGGTGAGCG-3′ ERIC 2 (Ardakani and Ranjbar 2016; Ahmed et al. 2025b). The thermal conditions applied for ERIC-PCR are shown in Table 1. PCR products were run on 2% agarose at 90 V for 2.5 h, and the bands were visualized using gel imaging via UV imaging system device (SYNGENE G:Box, UK).

**Table 1:** ERIC-PCR and RT-qPCR conditions

ERIC-PCR		RT-qPCR	
**Step**	**Temperature (°C)/Time**	**Step**	**Temperature (°C)/Time**
Pre-denaturation	94 °C/1 min	Pre-denaturation	95 °C/5 min
Amplification (30 Cycles)	94 °C/30 s	Amplification (40 cycles)	95 °C/10 s
	52 °C/35 s		55 °C/15 s
	72 °C/4 min		72 °C/20 s
Final Extension	72 °C/5 min	Melting	95 °C/5 s
			60 °C/1 min
			95 °C/–
		Final Extension	72 °C/2 min
		Cooling	40 °C/10 s

### Detection of biofilm formation with spectrophotometric microplate method and crystal violet (CV) staining

The biofilm ability of *E. coli* strains (n = 24) was quantified with the spectrophotometric microplate method including crystal violet (CV) staining (Aydin et al. 2024). Urinary *E. coli* isolates were first plated on Mueller–Hinton Agar (MHA) medium (Merck, Darmstadt, Germany) and incubated at 37 °C for 20–24 h. Then bacterial suspensions were prepared in tryptic soy broth with 2.5% glucose (TSBG, Oxoid, UK) medium (3 mL) and adjusted to 0.5 McFarland turbidity standard**.** Following the addition of TSBG (180 μL) medium to the wells, the inoculum suspension (20 μL) was seeded. The microplates were incubated at 37 °C for 24 h to be able to form biofilm. The well contents were gently aspirated and washed with sterile phosphate-buffered saline (Oxoid, UK) to remove non-adherent cells. The microplates were allowed to air dry at room temperature. Methanol (200 μL) was added to each well to adhere the microorganisms. After 15 min, crystal violet (2%, 200 μL) solution was added to each well to stain them after the methanol was aspirated. The contents of the well were disposed of after 15 min. The microplates were washed with tap water until the rinse water was colourless. After drying, 200 μL of 95% ethanol was added to the wells, and the plates were incubated for 10 min. The optical density (OD) values of the wells were measured at 570 nm, by using spectrophotometric microplate reader (CLARIOstar Plus Microplate Reader, BMG LabTech, Cary NC, USA). The OD of the wells with just TSBG medium was employed as negative control. Three standard deviations over the mean OD of the negative controls (only containing medium) were the definition of the cut-off optical density, or OD_C_. Each measurement was run in triplicate. GraphPad Prism 5.03 (San Diego, CA, USA) was used for statistical analyses (t-test, One-way ANOVA), with *p* < 0.05 being deemed significant. The biofilm forming capacity of isolates was categorized according to the following criteria: OD ≤ OD_C_: no biofilm, OD_C_ < OD ≤ (2 × OD_C_): weak biofilm, (2 × OD_C_) < OD ≤ (4 × OD_C_): moderate biofilm, and (4 × OD_C_) < OD: strong biofilm (Temel and Erac 2022).

### Determination of antimicrobial activity for EMP and MET

The broth microdilution method was performed in accordance with EUCAST (European Committee on Antimicrobial Susceptibility Testing) criteria to detect minimum inhibitory concentrations (MICs) of EMP and MET against five representative *E. coli* strains (E15, E18, E24, E34, and E37) (EUCAST v.16.0, 2026). Initially, each strain was streaked onto MHA and incubated overnight to obtain fresh colonies. Subsequently, inoculum suspensions to a turbidity standard of 0.5 McFarland were prepared in physiological saline (0.9% NaCl) using the direct colony suspension method. All bacterial suspensions were diluted 1/100 using Mueller–Hinton Broth medium before use to adjust to 5 × 10^5^ CFU/mL. After adding Mueller–Hinton Broth (50 µL), the drug solution (50 µL) was added to the first well, and serial dilutions were prepared by using the next wells. Then, the inoculum suspensions were added, and the microplates were incubated for 20–24 h at 37 °C (Ahmed et al. 2025a). At the end of the incubation period, the turbidity in the wells was assessed visually, and the lowest concentrations that did not show bacterial growth were recorded as the MIC of the drug (EUCAST v.16.0, 2026).

### Checkerboard assays

The combined efficacy of MET and EMP was evaluated with the checkerboard method. Five clinical *E. coli* isolates (E15, E18, E24, E34, and E37) known to be from different clones were selected as representatives using ERIC-PCR results (Supplement Fig. 3). Representative isolates were first grown by incubating on MHA at 37˚C for 18–20 h. Bacterial suspensions prepared from fresh cultures (in 0.9% NaCl) were adjusted to a 0.5 McFarland and diluted (1/100). Five different concentrations of EMP and MET solution, including 2xMIC, MIC, MIC/2, MIC/4, and MIC/8, were prepared and combined in the microplates. Finally, 100 μL of bacterial suspension was added, and the microplates were incubated at 37˚C for 16–24 h. The types of interaction between drugs were evaluated based on the fractional inhibitory concentration index (FICI) and the fractional inhibitory concentration (FIC) values for each combination. The following formulae were used to calculate the FIC index:

$$\it \it \mathrm{F}\mathrm{I}\mathrm{C} \ {d}\mathrm{r}\mathrm{u}\mathrm{g} \mathrm{A}: ({MIC}_{\mathrm{d}\mathrm{r}\mathrm{u}\mathrm{g} \mathrm{A} \ {i}\mathrm{n} \ {c}\mathrm{o}\mathrm{m}\mathrm{b}\mathrm{i}\mathrm{n}\mathrm{a}\mathrm{t}\mathrm{i}\mathrm{o}\mathrm{n}})/(\mathrm{M}\mathrm{I}\mathrm{C} \mathrm{d}\mathrm{r}\mathrm{u}\mathrm{g} \mathrm{A} \ {a}\mathrm{l}\mathrm{o}\mathrm{n}\mathrm{e})$$

$$\it \it \mathrm{F}\mathrm{I}\mathrm{C} \ {d}\mathrm{r}\mathrm{u}\mathrm{g} \mathrm{B}: ({MIC}_{\mathrm{d}\mathrm{r}\mathrm{u}\mathrm{g} \mathrm{B} \ {i}\mathrm{n} \ {c}\mathrm{o}\mathrm{m}\mathrm{b}\mathrm{i}\mathrm{n}\mathrm{a}\mathrm{t}\mathrm{i}\mathrm{o}\mathrm{n}})/(\mathrm{M}\mathrm{I}\mathrm{C} \mathrm{d}\mathrm{r}\mathrm{u}\mathrm{g} \mathrm{B} \ {a}\mathrm{l}\mathrm{o}\mathrm{n}\mathrm{e})$$

$$FIC index (FICI): ({FIC}_{drug A})+({FIC}_{drug B})$$

Synergistic, partial synergy, additive effect, indifferent, and antagonist interactions were defined by FICI values of ≤ 0.5, 0.5 to 0.75, 0.75 to 1, 1 to 4, and > 4, respectively.

### Determination of antibiofilm activity of EMP and MET *via *crystal violet staining

Considering the broth microdilution results, the antibiofilm activities of antidiabetic drugs on five representative *E. coli* isolates at subinhibitory (MIC/2 and MIC/4) concentrations were investigated using spectrophotometric methods. The spectrophotometric microplate method including CV staining was performed with the steps mentioned above. The biofilm-forming capacity of isolates in the presence and absence of drugs was calculated by averaging the OD values measured in replicate runs. The impact ratios on biofilm formation (IRB) of these antidiabetic drugs were calculated according to the formula:

$$Impact \ Ratio \ on \ Biofilm\ (IRB) (\%) = ({OD}_{A}-{OD}_{B})/{OD}_{A}\times 100$$

$$({OD}_{A}: Optical \ density \ of \ the \ well \ containing \ bacterial \ inoculum \ and \ medium)$$

$$({OD}_{B}: Optical \ density \ of \ the \ well \ containing \ the \ drug)$$

### Investigation of the effects of EMP and MET on virulence-associated genes

The presence of *fimH*, *luxS*, and *rpoC* genes in the representative isolates was confirmed by conventional PCR. The effects of EMP and MET on virulence-associated genes in *E. coli* isolates were investigated by real-time quantitative polymerase chain reaction (RT-qPCR). Virulence-associated genes *fimH*, and *luxS* which are frequently related to biofilm formation and antibiotic resistance in *E. coli* isolates (E15, E18, E24, E34, and E37), were selected as target genes. The *polB* and *rpoC* genes were included as housekeeping genes. The primer sequences used for both conventional PCR confirmation and RT-qPCR expression analyses were listed in Table 2.

**Table 2:** Primers for conventional PCR and qPCR

	**Gene**	**Primers**	**Product size (bp)**	**References**
***Conventional PCR***	***fimH***	(F) GGGAACCATTCAGGCAGTG	1037 bp	designed for this study
		(R) GGGCTAACGTGCAGGTTTTT		
	***luxS***	(F) TCTTCTGGATGTCAGCCGGGAT	741 bp	designed for this study
		(R) CAGACTCGCCTGGGAAGAAAG		
	***rpoC***	(F) GGGCTAACGTGCAGGTTTTT	4279 bp	designed for this study
		(R) GGGCTAACGTGCAGGTTTTT		
	***polB***	(F) CCCGCCATCAGTTTGTCGAT	232 bp	designed for this study
		(R) GCCGTTTTCTGATGCCAACC		
***qPCR***	***fimH***	(F) GTGCCAATTCCTCTTACCGTT	164 bp	Hojati et al. 2015
		(R) TGGAATAATCGTACCGTTGCG		
	***luxS***	(F) GCTTCCTGCAACGAGTGCAT	112 bp	Al-Bayati and Samarasinghe 2022
		(R) ATGCCTGGAAAGCGGCAATG		
	***polB***	(F) TCAGGAGTACGTCCGCGAAA	150 bp	designed for this study
		(R) TCTTCATCGGCAAGGCGAGC		
	***rpoC***	(F) GATCGCGGGAGTAAGTTGCA	182 bp	designed for this study
		(R)GACGTATACCGTCTGCAGGG		

*E. coli* suspensions were exposed to the sub-inhibitory concentrations (MIC/2 and MIC/4) of EMP and MET, before the RNA isolation. Test tubes containing bacterial culture and active substance were incubated in a shaking incubator at 37 °C and 200 rpm to ensure exposure to the drugs. At the 18th hour, total RNA isolation was performed from samples using a commercial total RNA purification kit (GMbiolab, Taiwan). The concentrations of RNA isolates were measured using the Beckman Coulter DU-730 device (California, USA) and stored at −80 °C. Total RNA was quantified spectrophotometrically, and all samples were standardized to a concentration of 1 μg per 20 μL reaction using the NanoDrop spectrophotometer for cDNA synthesis. Onescript Plus Reverse Transcriptase cDNA Synthesis Kit (ABM, USA) was used, and the reaction mixture containing the following was prepared: total RNA (10 μL), primer (1 μL), dNTP mix (10 mM, 1 μL), and nuclease-free water 4.5 μL. The mixture was kept in the thermocycler at 65 °C for 5 min, then kept on ice (1 min), and the following were added to the mixture: 5X RT Buffer (4 μL), RNase OFF ribonuclease inhibitor (0.5 μL), and Onescript Plus RTase (1 μL). The mixture (20 μL) was placed into a thermal cycler, and cDNA synthesis was performed (25 °C/10 min, 50 °C/15 min, 85 °C/5 min). The synthesized cDNAs were kept at −20 °C.

### Real time-quantitative polymerase chain reaction (RT-qPCR)

RT-qPCR experiments with cDNAs used the 2X Magic SYBR Mix (Procomcure, Australia) kit. cDNA amounts were measured and adjusted to equal concentrations, then 2 μL were added to 96-well Armadillo (Thermo Scientific, USA) microplates for RT-qPCR. The components and amounts of the mixture for the RT-qPCR reaction were as follows: SYBR Mix (10 μL), forward primer (10 μM, 1 μL), reverse primer (10 μM, 1 μL), and nuclease-free water (6 μL). After adding the master mix to each cDNA-containing well, the microplate was sealed and centrifuged at 500xg (for 1 min). The RT-qPCR was performed using LightCycler 480 II (Roche, Switzerland), according to the conditions in Table 1. Results were evaluated with the Delta-Delta CT (ΔΔCT) method, taking into account the threshold cycle (CT) values obtained from the LightCycler 480 Software release 1.5.0. SP3 software. The qPCR step was performed in triplicate. The following formulas were used to interpret the experimental results:

$$\Delta CT \left(sample\right)= CT \left(target\right)-CT (reference)$$

$$\Delta \Delta CT = \Delta CT \left(Exposed \ sample\right)-\Delta CT (control \ sample)$$

$$Expression \ Fold \ Change = {2}^{-(\Delta \Delta CT)}$$

### Statistical analyses

All experimental assays, including MIC determinations, biofilm quantification, and RT-qPCR, were performed in three independent biological replicates, each conducted with technical triplicates to ensure reproducibility. Statistical analyses were conducted using GraphPad Prism version 8.0 (San Diego, CA, USA). Data were presented as the mean ± standard deviation (SD). The normality of the data distribution was verified using the Shapiro–Wilk test. Significant differences between the untreated control and the drug-treated groups (at various sub-MICs) for biofilm inhibition assays and gene expression levels were determined using One-way Analysis of Variance (ANOVA) followed by Dunnett’s post-hoc test for multiple comparisons. In the synergy assays, fractional inhibitory concentration index (FICI) values were calculated to categorize the interactions. For RT-qPCR data, the relative fold changes in gene expression were calculated using the 2^−ΔΔ*CT*^ method. A *p*-value of less than 0.05 was considered to indicate a statistically significant difference. (*p < 0.05, **p < 0.01, ***p < 0.001).

## Results

### Identification and antibiotic susceptibility profiles of clinical *E. coli* isolates

Clinical *E. coli* isolates were identified using VITEK-2 and BD Phoenix™ M50 ID/AST (Becton Dickinson, Maryland, USA) automated systems*.* The antibiotic susceptibility profiles of twenty-four clinical *E. coli* isolates are listed in Supplement Table 1. The significant growth thresholds for *E. coli* isolates according to the NHS (National Health Service, UK) and UK Standards for Microbiology Investigations (UK SMI) were considered for verifying whether it is a uropathogenic strain in this study (NICE 2023). Considering the NHS guidelines and interpreting criteria, clinical *E. coli* isolates whose growth threshold values were ≥ 10^3^ CFU/mL were selected for this study. The antibiotic susceptibility profiling of forty *E.coli* isolates revealed significant resistance levels to commonly used antimicrobials. The highest resistance rates were observed for ampicillin (65%) and cefuroxime (65%), followed by ciprofloxacin (32.5%) and trimethoprim/sulfamethoxazole (27.5%). Notably, all isolates were susceptible to carbapenems (imipenem, meropenem, and ertapenem), amikacin, and fosfomycin (0% resistance). Based on the criteria of resistance to at least three different antimicrobial classes, 40% (16/40) of the isolates were identified as Multidrug-Resistant (MDR).

### Clonal relationships

The forty urinary *E. coli* isolates were categorized into 12 distinct clones based on their molecular fingerprinting profiles obtained via ERIC-PCR (Supplement Fig. 3). Representative isolates with strong biofilm-forming capacity, such as E15, E18, E24, E34, and E37, were selected from these unique genetic backgrounds for subsequent antimicrobial and gene expression analyses to ensure a non-clonal and representative study population. The transformation of the bands in the gel was assessed after ERIC-PCR was performed with appropriate primers targeting repetitive gene regions. The clonal relationships of 40 clinical *E. coli* isolates were determined using the Weighted Pair Group Method with the Arithmetic Mean method using FigTree v1.4.4 and are shown in the dendrogram as Fig. 1. A total of 40 isolates were distributed across 12 distinct clones. Among them, 24 isolates from different clones were investigated for biofilm detection, and then five representative strains (E15, E18, E24, E34, and E37), which were strong biofilm producers, were included in gene expression analyses.

### Biofilm capacity of *E. coli* isolates

**Fig. 2:**
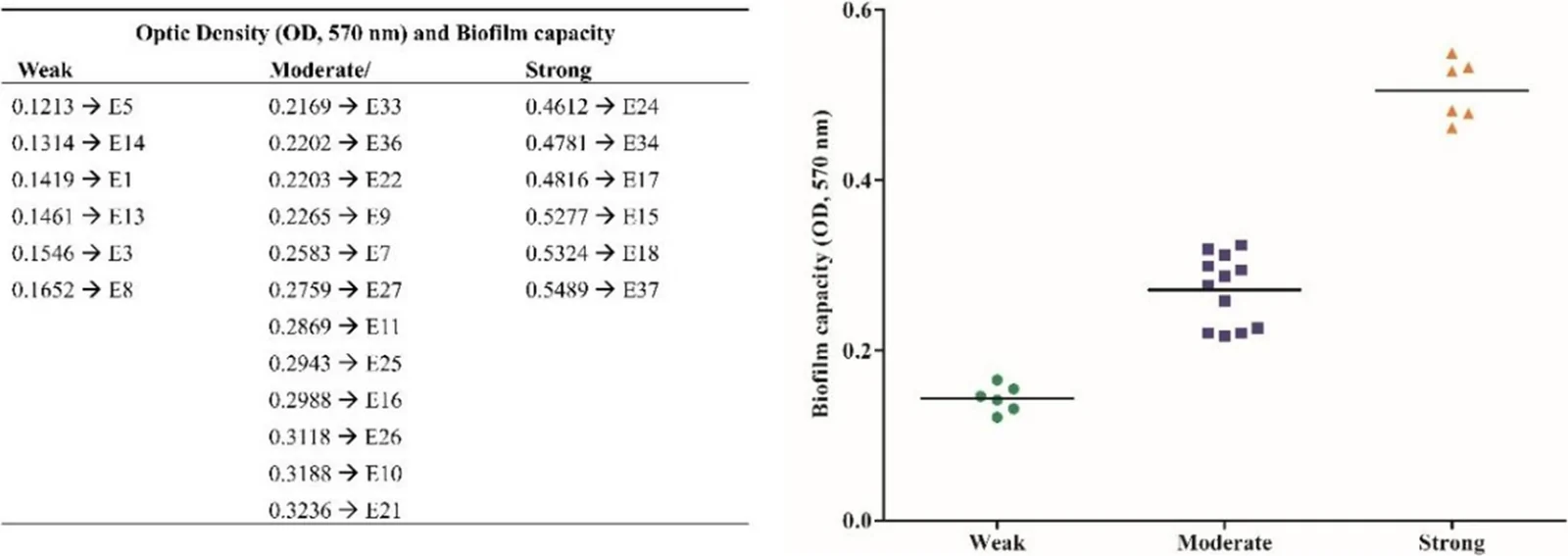
Biofilm capacity of urinary *E. coli* isolates. OD: Optical Density, OD_C_: Optical density of biofilm control well (containing bacterial inoculum and medium), OD_C_: 0.1065

Biofilm capacity of twenty-four *E. coli* isolates was presented in Fig. 2. Six isolates were determined to be weak biofilm producers, 12 to be intermediate, and 6 to be strong biofilm producers.

### Antimicrobial activity of EMP and MET

The MIC values for EMP and MET are shown in Table 3. The MIC values of EMP were detected as lower than MET, ranging from 3.12 to 6.25 mg/mL. The FICI values obtained for the MET + EMP combination against isolates E15, E18, E24, E34, and E37 were calculated as 0.8, 0.6, 1.1, 1.2, and 0.4, respectively. For the combination of MET + EMP, partial synergy was detected in isolate E18 (FICI: 0.6), and synergy was observed for E37 (FICI: 0.4).

**Table 3:** Minimum inhibitory concentrations (MICs) of empagliflozin (EMP) and metformin (MET) against five representative *E. coli* isolates

ERIC-PCR Clone	Isolate	MIC_MET_ (mg/mL)	MIC_EMP_ (mg/mL)
D	**E15**	25	3.125
I	**E18**	50	3.125
E	**E24**	50	6.250
C	**E34**	25	3.125
B	**E37**	50	3.125

### Antibiofilm activity of EMP and MET

The antibiofilm activities of EMP and MET were investigated at subinhibitory concentrations (MIC/2 and MIC/4). The potential antibiofilm activity of drugs was interpreted based on changes in measured optical density (OD) values and calculations. OD and IRB values in the presence/absence of these drugs were presented in Table 4.

**Table 4:** Optical density and impact ratio on biofilm formation (IRB) of empagliflozin (EMP) and metformin (MET) at sub-minimum inhibitory concentrations (MIC/2 and MIC/4)

		**MET**					**EMP**				
**Clone**	**Iso**	**OD**_**C**_	**OD**_**MIC/2**_	**IRB** _**MIC/2**_	**OD**_**MIC/4**_	**IRB** _**MIC/4**_	**OD**_**C**_	**OD**_**MIC/2**_	**IRB** _**MIC/2**_	**OD**_**MIC/4**_	**IRB** _**MIC/4**_
D	**E15**	0.529 ± 0.089	0.342 ± 0.045*	35.46 ↓	0.390 ± 0.073*	26.34 ↓	0.479 ± 0.099	0.266 ± 0.045**	44.52 ↓	0.316 ± 0.064*	33.95 ↓
I	**E18**	0.495 ± 0.017	0.149 ± 0.040***	69.83 ↓	0.155 ± 0.029**	28.18 ↓	0.526 ± 0.054	0.198 ± 0.050***	62.32 ↓	0.275 ± 0.046**	28.62 ↓
E	**E24**	0.513 ± 0.018	0.188 ± 0.013***	63.38 ↓	0.166 ± 0.022 (ns)	9.19	0.520 ± 0.085	0.245 ± 0.035**	52.93 ↓	0.196 ± 0.031**	43.12 ↓
C	**E34**	0.585 ± 0.024	0.257 ± 0.050**	56.07 ↓	0.266 ± 0.046**	20.24 ↓	0.493 ± 0.065	0.210 ± 0.016**	37.06 ↓	0.313 ± 0.037**	36.33 ↓
B	**E37**	0.531 ± 0.103	0.256 ± 0.083**	51.80 ↓	0.257 ± 0.055**	52.86 ↓	0.508 ± 0.085	0.137 ± 0.006***	72.94 ↓	0.191 ± 0.032**	42.71 ↓
	***E. f***	0.554 ± 0.073	0.348 ± 0.088*	37.20 ↓	0.504 ± 0.041 (ns)	9.04	0.554 ± 0.046	0.434 ± 0.072*	21.60 ↓	0.450 ± 0.043*	18.87 ↓

Iso.: Isolate, *E.f*.: *Enterococcus faecalis,* OD_C_: Optical density of biofilm control well (containing bacterial inoculum and medium), OD_MIC/2_: Optical density of the well containing bacterial inoculum, medium and drug (at MIC/2), OD_MIC/4_: Optical density of the well containing bacterial inoculum, medium and drug (at MIC/4), IRB: Impact ratio on biofilm formation, EMP: Empagliflozin, MET: Metformin, MIC: Minimum inhibitory concentration, ± SD: Standard deviation, (ns): Not statistically significant (p > 0.05). Significance levels: *p < *0.05*, **p < *0.01*, ***p < *0.001* compared to the untreated control (OD_C_). Symbols: (↓) indicates a reduction in biofilm formation

### Detection of virulence-associated genes

**Fig. 3:**
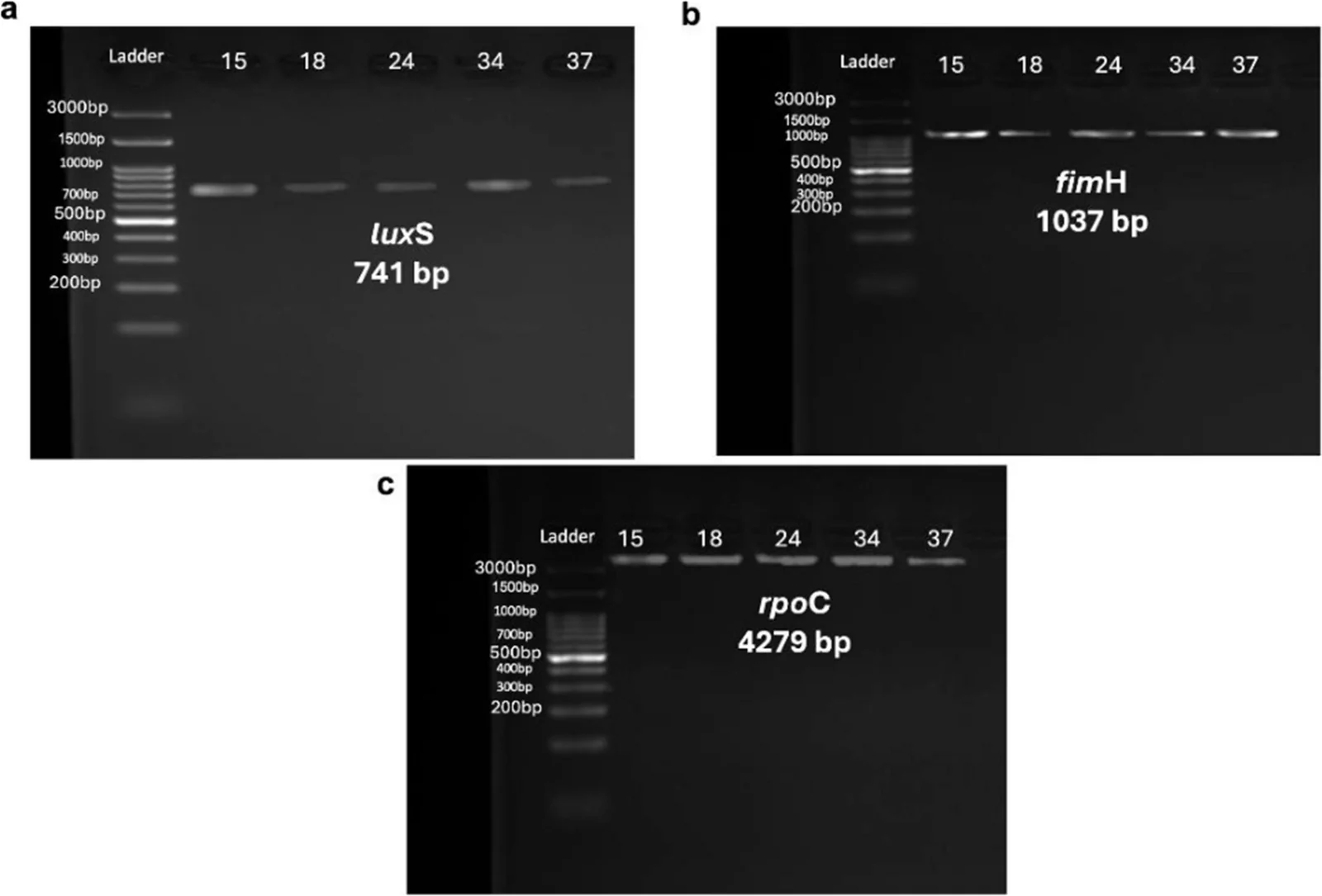
Gel electrophoresis images of the *luxS* (a), *fimH* (b), and *rpoC* (c) genes in *E. coli* isolates. **(a)** Amplification of the *luxS* gene (741 bp), **(b)** amplification of the *fimH* gene (1037 bp), and **(c)** amplification of the *rpoC* gene (4279 bp). Lane M: DNA ladder, Lanes 15–37: Clinical urinary *E. coli* isolates

The *fimH, luxS*, and *rpoC* genes were investigated in representative *E. coli* isolates by PCR. The gel images shown in Fig. 3 indicate that the representative strains have *fimH, luxS*, and *rpoC* genes.

### Effect of EMP and MET on virulence-associated gene expression levels

**Fig. 4:**
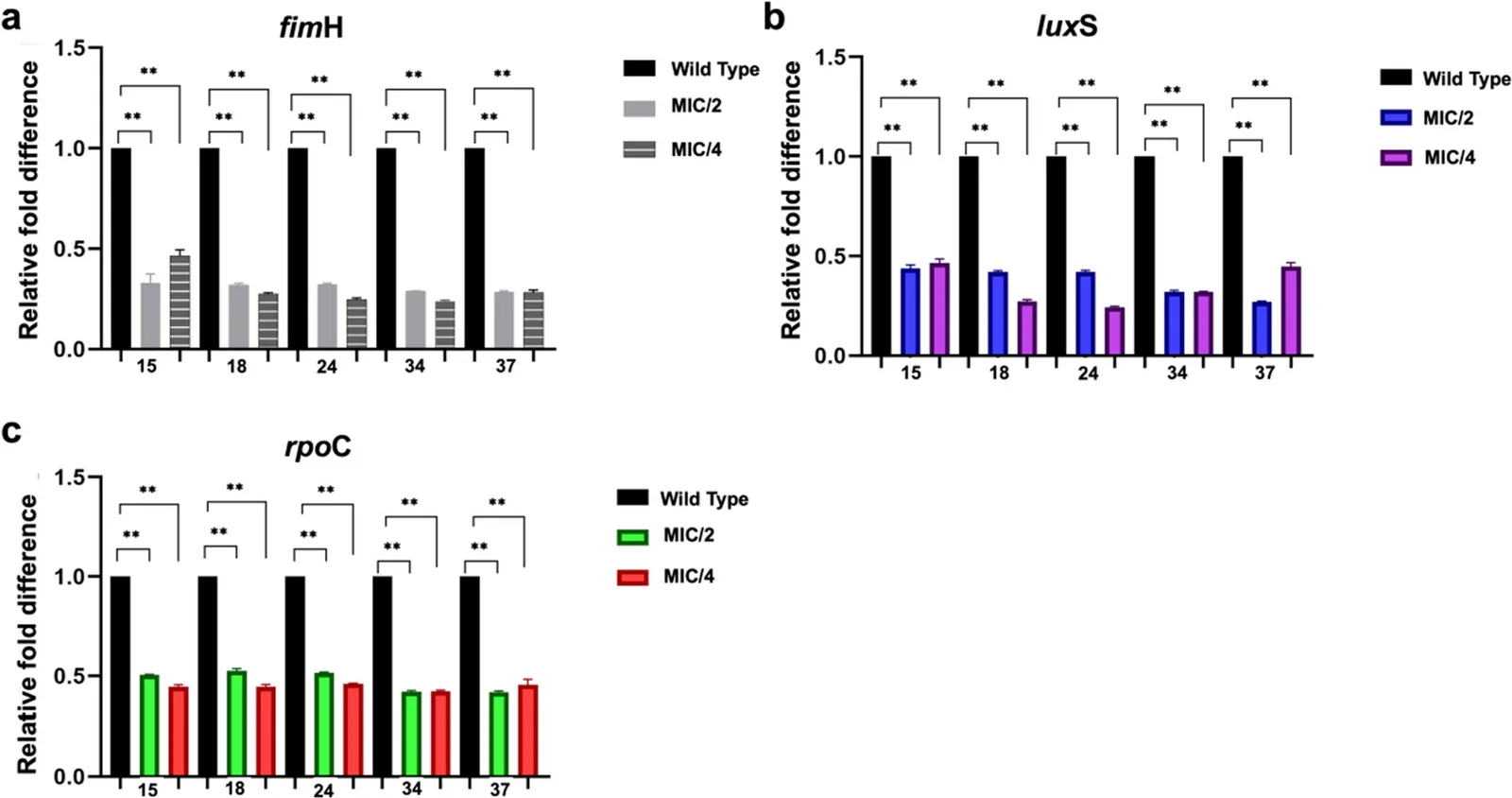
Effect of MET on *fimH, luxS*, and *rpoC* gene expression levels at sub-MICs. **(a)** Expression of *fimH* gene, **(b)** expression of *luxS* gene, and **(c)** expression of the housekeeping gene *rpoC*. Statistical significance is indicated by asterisks (*p < 0.05, **p < 0.01)

Considering the gene expression analyses, MET reduced *fimH* gene expression approximately 69.1% in five strong biofilm producer isolates at MIC/2, while this ratio decreased to 69.9% inhibition at MIC/4. After exposing metformin at MIC/2 and MIC/4 concentrations, *luxS* gene expression was reduced by 53.5–75.7% in the five isolates (Supplement Table 2). The relative fold difference in gene expression levels in the presence of MET is presented in Fig. 4.

**Fig. 5:**
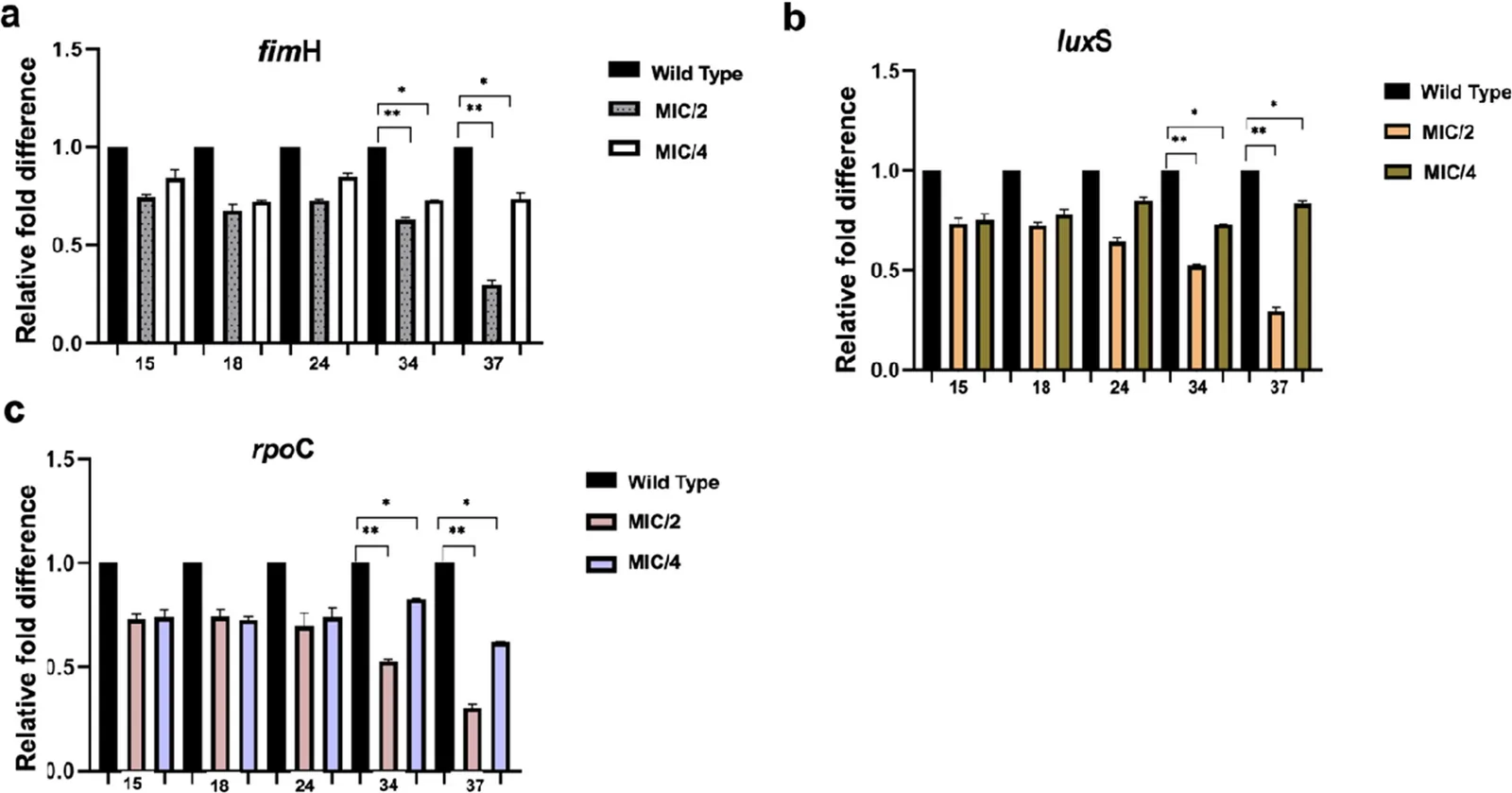
Effect of EMP on *fimH, luxS,* and *rpoC* expression at sub-MICs. Effect of EMP on *fimH, luxS*, and *rpoC* gene expression levels at sub-MICs. **(a)** Expression of *fimH* gene, **(b)** expression of *luxS* gene, and **(c)** expression of the housekeeping gene *rpoC*. Statistical significance is indicated by asterisks (*p < *0.05*, **p < 0.01)

The most dramatic expression change following EMP exposure was observed for isolate 37. EMP was shown to reduce gene expression by 70.4, 70.6, 69.6% at MIC/2 concentration in the *fimH*, *luxS*, and *rpoC *genes in this isolate, respectively. After exposing EMP at MIC/2 and MIC/4 concentrations, *luxS* gene expression was reduced by 14.8–70.6% in the five isolates. The *rpoC* gene was observed to have a decreased gene expression level by 17.1–69.6% in the presence of subinhibitory EMP concentrations (Supplement Table 3). In five isolates, subinhibitory EMP concentrations downregulated *fimH* transcription, with inhibition rates of an average 38.4% at MIC/2 and 22.4% at MIC/4. The variations in the gene expression levels with EMP were presented in Fig. 5. Detailed amplification efficiency data for the primers were provided in Supplement Table 4.

**Fig. 6:**
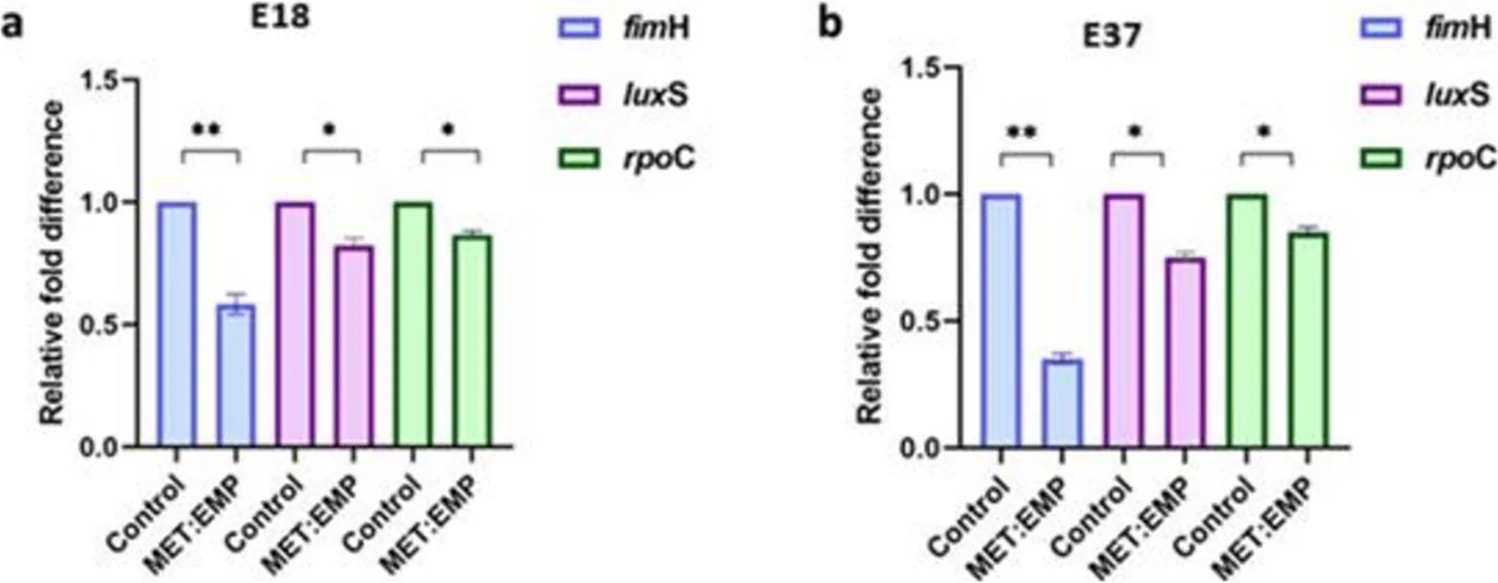
Effect of synergistic MET:EMP combinations on *fimH*, *luxS*, and *rpoC* expression in (**a**) Isolate E18 and (**b**) Isolate E37. (*p < *0.05*, **p < 0.01)

Considering the findings of checkerboard assays, biofilm-associated gene expressions in E18 and E37 were evaluated following exposure to synergistic MET:EMP combinations using RT-qPCR. For E18, the synergistic MET:EMP combination (MET: 5 mg/mL; EMP: 1.56 mg/mL) resulted in a downregulation of *fimH*, *luxS*, and *rpoC* gene expression by 41.6%, 17.6%, and 13.3%, respectively (Fig. 6a). For E37, the synergistic MET:EMP combination (MET: 7.5 mg/mL; EMP: 1.56 mg/mL) led to a reduction in gene expression of 65% for *fimH*, 25% for *luxS*, and 15% for *rpoC* (Fig. 6b).

## Discussion

Antimicrobial resistance (AMR), called a 'pandemic', is leading to dramatic morbidity and mortality rates. New strategies for combating infectious diseases are needed to protect both public health and healthcare investments, and drug repurposing is a prominent approach. In the current era, research focusing on the antimicrobial properties of non-antibiotics is attracting global attention. Considering the association between diabetes mellitus and infectious diseases, antidiabetic drugs have emerged as a prominent area of interest in drug repurposing research, particularly with regard to their potential antimicrobial properties (Haobus et al. 2021). SGLT-2 inhibitors are a novel class of antihyperglycemic drugs for T2DM, which inhibit renal glucose reabsorption, causing glucosuria and lowering blood glucose levels. However, urinary glucose excretion may be considered a risk factor for urinary tract infections (UTIs). Nevertheless, meta-analyses evaluating clinical data have suggested that the risk of UTIs associated with SGLT-2 inhibitors is still controversial (Zaccardi et al. 2016; Li et al. 2017; Liu et al. 2017; Uitrakul et al. 2022; Kittipibul et al. 2024). A comprehensive meta-analysis reported no difference in risk of UTIs between SGLT-2 inhibitors and the control group (Liu et al. 2017). Similarly, the results of another meta-analysis indicated that there was no increased risk of UTIs in patients using SGLT-2 inhibitors other than dapagliflozin (Li et al. 2017). In contrast, Zaccardi et al. emphasized that the dapagliflozin group exhibited a heightened risk of UTIs when compared to the control and empagliflozin groups (Zaccardi et al. 2016). A potential explanation for these inconsistent clinical findings regarding the relationship between SGLT-2 inhibitors and UTIs may be the frequent concomitant use of these drugs with metformin in clinical practice and the possible antimicrobial activity of metformin (Malik et al. 2018). The analysis of UTI adverse events reported to the FAERS database revealed that the combination of SGLT-2 inhibitors and metformin resulted in fewer reported UTI adverse events (Aksoyalp and Temel 2024). Furthermore, the study identified that canagliflozin and dapagliflozin demonstrated a higher reporting frequency for UTIs in clinical safety databases compared to empagliflozin (Aksoyalp and Temel 2024). However, the potential antimicrobial properties of SGLT-2 inhibitors, both as monotherapy and in combination with other pharmaceutical agents, are still to be elucidated. Therefore, the present study focused on the investigation of the antivirulence and antibiofilm activity of empagliflozin, both alone and in combination with metformin. In particular, to the best of our knowledge, our present study is the first to report results regarding the antimicrobial and antibiofilm effects of empagliflozin against urinary *E. coli* isolates.

Firstly, empagliflozin showed in vitro antimicrobial activity against *E. coli* isolates with MICs ranging from 3.12 to 6.25 mg/mL in the present study. Among the SGLT-2 inhibitors, only canagliflozin has recently been the focus of research in order to determine its antimicrobial effects against methicillin-resistant *Staphylococcus aureus* (Gu et al. 2023). Moreover, the combination of canagliflozin with penicillin has been observed to result in approximately threefold increased antibacterial activity (Gu et al. 2023)*.* Similar to the findings of our study, canagliflozin had significant antibacterial activity against *E. coli* as well as *S. aureus* and *P. aeruginosa* (Li et al. 2024). Another intriguing finding of the present study was the observation that the MIC values of empagliflozin were found to be lower than those of metformin. Additionally, the synergistic interaction observed for empagliflozin combination with metformin against two *E. coli* isolates (E18 and E37) was also a noteworthy result. While the combination of MET and EMP showed additive effects in certain isolates like E18, the corresponding downregulations in gene expression (13–17%) were relatively minor. We acknowledge that such modest transcriptional changes may not translate into a biologically meaningful impact on the phenotype of the bacteria in a clinical or physiological context. These findings relevant to the antimicrobial effect potential of empagliflozin may provide a possible basis for understanding the inconsistent results concerning the association between SGLT-2 inhibitors and UTIs. However, it is important to note that the synergistic interaction between empagliflozin and metformin was observed in a limited number of isolates, suggesting that this effect may be strain-dependent. While these preliminary findings are encouraging, the clinical significance of this combination therapy remains to be fully elucidated through further investigations involving a larger and more diverse collection of clinical UPEC strains.

Metformin has been suggested to exhibit antimicrobial activity by limiting bacterial proliferation caused by hyperglycemia for these infections (Garnett et al. 2013; Patkee et al. 2016; Malik et al. 2018). Metformin has been shown to increase susceptibility to doxycycline and minocycline in multidrug-resistant *S. aureus, E. faecalis*, *E. coli,* and *Salmonella enteritidis* strains. Therefore, metformin may be a novel tetracycline adjuvant to improve treatment outcomes in resistant infections (Liu et al. 2020). In addition, Liu et al. reported that in vitro metformin administration has the potential to boost the immune response and may alleviate the inflammatory process (Liu et al. 2020). Masadeh et al. studied the potential effect of metformin on antibiotics against methicillin-resistant *S. aureus* and *P. aeruginosa* (Masadeh et al. 2021). The highest and lowest antibiotic MIC reduction was 93% for chloramphenicol MIC and 75% for levofloxacin MIC according to FIC values obtained from checkerboard assays (Masadeh et al. 2021). In another study, metformin, in combination with doxycycline, has been found to reduce antibiotic doses for *E. coli* and *Shigella boydii* (Hossain et al. 2024). The MIC values of metformin against five representative *E. coli* isolates were found to range from 25 to 50 mg/mL in this study. Synergistic interaction was detected for empagliflozin and metformin in two strains. Considering the findings of studies involving various bacterial species and antibiotics with different chemical structures or mechanisms of action, metformin appears to have antimicrobial activity; however, its synergistic effect with antibiotics has not been definitively determined. The precise mechanism of antibacterial activity of metformin reported in previous in vitro studies is not yet fully understood. Further molecular studies, including molecular docking and dynamic simulations, are needed to better elucidate its antibacterial activity. In this context, Hossain et al. performed molecular docking research to detect interactions between metformin and critical bacterial proteins, MexA and AcrB (Hossain et al. 2024). Notably, metformin has a strong binding affinity to AcrB and MexA and has an inhibitory effect on AcrB. Metformin-mediated inhibition of AcrB, an important efflux transporter protein, may be an effective mechanism to increase susceptibility to some antibiotics in resistant strains (Hossain et al. 2024). Another theory suggests that the antibacterial activity of metformin is due to its interaction with the outer membrane of the bacteria (Nikaido 2003; Liu et al. 2020; Li et al. 2023). It is hypothesised that metformin may damage the integrity of the outer membrane by displacing the divalent cations, such as Mg^2+^, that play a stabilising role in the bacterial outer membrane (Ruiz et al. 2006). Metformin may interact with the cytoplasmic membrane in Gram‐positive and Gram‐negative bacteria (Liu et al. 2020). UPECs with biofilm-forming capacity can become more resistant to antibiotics within the biofilm (Behzadi et al. 2020). Initial attachment of bacteria to host cells via surface adhesions is a pivotal step in forming biofilm. Numerous studies have highlighted differences in fimbriae gene distribution between strong and weak biofilm-forming bacteria (Whelan et al. 2023). The adhesion and quorum sensing (QS) signalling systems are both major and complex factors that play an important role in UPEC for bacterial colonisation. Hence, the present study focused on the antibiofilm potential of metformin and empagliflozin. It was also investigated the effects of these drugs on the fimbria and QS system-associated genes in *E. coli*. It should be noted that the crystal violet assay measures the total biofilm biomass, encompassing the cellular components and the extracellular polymeric matrix. To distinguish between true antibiofilm activity and general growth inhibition, we evaluated the effects at sub-MIC levels. The significant reduction in biofilm formation at concentrations that do not suppress planktonic growth (MIC/2 and MIC/4) suggests that empagliflozin and metformin specifically target the mechanisms of biofilm development and structural integrity in UPEC isolates. Metformin at MIC/2 could reduce biofilm formation in urinary *E. coli* isolates by 35.3–69.8%. Similar to our results, antibiofilm effects of metformin on *Streptococcus suis, E. coli,* and *P. aeruginosa* have been reported in different studies (Ye et al. 2021; Jiang et al. 2023; Zuo et al. 2023). In addition, empagliflozin inhibited bacterial biofilm formation by 37.0–72.9%. A study revealed that canagliflozin disrupts bacterial biofilm formation in methicillin-resistant *S. aureus* (Gu et al. 2023). In a different study, canagliflozin was shown to have a weak antibiofilm effect on *E. coli* and *P. aeruginosa*, while the antibiofilm effect on *S. aureus* was not significant (Li et al. 2024). The difference in the results of the antibiofilm effects of empagliflozin and canagliflozin on *E. coli* isolates may be attributable to the differences in the pharmacophore groups of these molecules (Lymperopoulos et al. 2021). The quantitative analysis presented in Table 4 highlights a significant phenotypic shift in the clinical *E. coli* isolates upon exposure to empagliflozin and metformin. The observed reduction in optical density values translates to a substantial impact ratio, with biofilm inhibition reaching up to 72.9% for certain isolates. This high level of biomass reduction is particularly significant as it suggests that these drugs do not merely slow down bacterial growth but actively interfere with the structural integrity of the biofilm matrix. Such a high impact ratio in a clinical setting could potentially enhance the penetration of conventional antibiotics and reduce the likelihood of persistence in the urinary tract.

Determining whether the antibiofilm effect seen in the phenotype has a genotypic background requires molecular gene analyses. QS systems, cell-to-cell communication systems, have been demonstrated to facilitate rapid adaptation of bacteria to changing environmental conditions and are implicated in bioluminescence, biofilm formation, virulence, antibiotic production, and competence in a variety of bacteria (Li and Nair 2012; Pereira et al. 2013). QS inhibition is the most prominent factor in providing antibiofilm effects (Abbas et al. 2017). The antimicrobial and antibiofilm effects of empagliflozin combined with metformin are thought to occur through QS signalling, and this inhibition of biofilm formation is determinative for bacterial colonization. The QS system encompasses the production and detection of autoinducers (AIs). *E. coli* produces biofilms in response to the small molecule autoinducer-2 (AI-2), a product of the *LuxS* enzyme. *LuxS,* an AI-2 synthesis-related gene, plays a key role in the QS process of *E. coli* and contributes to virulence (Pereira et al. 2013). The inhibitor effect of the empagliflozin and metformin on *fimH*, *luxS*, and *rpoC* virulence-associated genes expression in *E. coli* isolates was also evaluated to clarify the underlying mechanisms. The sub-MIC concentrations of empagliflozin and metformin caused a significant reduction in the expression of the virulence genes in the present study.

Previous studies on the antibacterial effect of metformin against *P. aeruginosa* species also suggest that the main mechanisms may be decreased QS and virulence gene expression, a strong relationship between metformin and QS receptors, compromised motility phenotypes, and inhibited exopolysaccharide production (Chadha et al. 2023). In parallel to our findings, Zuo et al. stated that metformin downregulates the biofilm-forming ability of *S. suis* and that metformin has a significant binding activity to *LuxS* protein and seems to be a suitable agent for control of *S. suis* (Zuo et al. 2023). Our RT-qPCR studies revealed that exposure to sub-MIC of metformin resulted in a significant reduction in *luxS* gene expression in *E. coli* isolates. In another study, metformin suppressed *fimH-1* by 42.5% in clinical *Klebsiella pneumoniae* isolates, and the antivirulence potential of metformin was attributable to its capacity to decrease expression of genes involved in QS. Ye et al. indicated that metformin inhibits flagellar motility and chemotaxis, which are vital for *E. coli* colonization (Ye et al. 2021). Jiang et al. showed that the promotion of bacterial surface aggregation by metformin was caused by an inhibition in swimming motility in *E. coli* (Jiang et al. 2023). In our study, we have shown preliminary data on the expression of the *fimH* gene associated with biofilm-forming urinary *E. coli* isolates. The product of this gene, *fimH* protein that constitutes Type I fimbriae, mediates initial adhesion of *E. coli* to urothelial cells, a critical step in bacterial colonization and biofilm production (Rodriguez-Miranda et al. 2024). In our study, exposure to sub-MIC concentrations of empagliflozin and metformin resulted in significant downregulation of *fimH* gene expression. The present findings suggest that the antimicrobial effects of empagliflozin and metformin may be linked to changes in the expression of these virulence-associated genes.

This study is pioneering in investigating the in vitro antimicrobial and antibiofilm effects of empagliflozin; however, there are some limitations to consider. Firstly, the findings are based on a relatively small sample size comprising a limited number of bacterial isolates, which may restrict the generalizability of these results. Secondly, the reduction in biofilm biomass was confirmed through crystal violet staining and genotypic analysis in this study. Future research incorporating metabolic activity assays such as XTT or visualization techniques like confocal laser scanning microscopy would provide further insights into the viability of the embedded bacterial cells. Lastly, a critical consideration in drug repurposing is the clinical achievability of effective in vitro concentrations. In the present study, the MIC values for empagliflozin (3.125–6.25 mg/mL) and metformin (25–50 mg/mL) were substantially higher than the peak plasma concentrations achieved with standard oral dosing (Cmax ≈ 0.310 µg/mL). While this disparity might initially suggest a challenge for systemic applications, the clinical potential of these agents should be evaluated through their unique pharmacokinetic profiles and their role as non-traditional antimicrobials. Both empagliflozin and metformin are primarily excreted via the renal system; empagliflozin, as an SGLT-2 inhibitor, specifically accumulates in the kidneys and reaches significantly higher localized concentrations in the urinary tract compared to systemic circulation. In pharmacokinetic studies of SGLT-2 inhibitors, the percentage of empagliflozin excreted unchanged in urine was found to range from 12.2% to 18.7% (Heise et al. 2013), which was reported to be higher than that of dapagliflozin and canagliflozin (< 3%) (Scheen 2014). More importantly, our findings demonstrate that these agents exert potent antibiofilm and anti-virulence effects at sub-inhibitory concentrations (MIC/2 and MIC/4), which are physiologically more relevant. The specific downregulation of key virulence markers such as *fimH* and *luxS* suggests that the observed effects are not a non-specific response to high concentrations, but rather a targeted interference with bacterial pathogenicity and QS mechanisms. Furthermore, both antibiofilm effects of these drugs and the synergistic interactions observed when these drugs are combined with conventional antibiotics point toward their role as adjuvants rather than standalone agents. By weakening the structural integrity of bacterial biofilms and suppressing adhesion factors, these drugs may be effective for antivirulans strategies for combating infections. Furthermore, the significant impact of sub-MIC concentrations on biofilm-related gene expression suggests that these drugs may serve as adjuvant therapies to impair bacterial virulence, even at lower doses. The present study was focused on urinary *E. coli* isolates and the effects of empagliflozin and metformin against other uropathogens remain to be elucidated. In addition, gene expression analysis was focused on a select number of virulence genes (*fimH, luxS,* and *rpoC*). Despite these limitations, to our knowledge, this is the first study to investigate the antivirulence and antibiofilm properties of empagliflozin against urinary *E. coli* isolates. These findings provide a basis for the design of more comprehensive in vitro and in vivo studies to further explore the repurposing potential of these medications. Despite the promising in vitro antibiofilm and antivirulence effects of empagliflozin observed in our study, it is essential to contextualize these findings within clinical practice. Recent studies have indicated that SGLT-2 inhibitors, including empagliflozin, are associated with an increased incidence of UTIs in diabetic patients, primarily due to induced glucosuria which provides a favorable environment for bacterial proliferation. Interestingly, these infections occur even in patients receiving concomitant metformin therapy. While our results suggest that these drugs can interfere with the adherence and QS mechanisms of *E. coli* at a molecular level, the overall clinical outcome is a complex balance between these inhibitory effects and the metabolic changes in the urinary environment. Therefore, our findings should be interpreted as a mechanistic insight into bacterial behavior rather than a definitive claim of clinical prophylaxis against UTIs. Future studies are warranted to evaluate the potential of these agents to inhibit initial bacterial adhesion and to degrade preformed, mature biofilms, which are critical factors in chronic and recurrent UTIs. Consequently, the present study has determined that empagliflozin exhibits antibacterial activity against urinary *E. coli* isolates and limits bacterial virulence by inhibiting biofilm formation. The main findings of this study are that the MICs of empagliflozin against urinary *E. coli* isolates are comparatively lower than those of metformin, and that there is a potential synergistic interaction between empagliflozin and metformin. The in vitro antibiofilm properties of empagliflozin and metformin may have the potential to prevent bacterial colonization of the urinary tract. The investigation of molecular mechanisms underlying the antimicrobial activities of SGLT-2 inhibitors and metformin could be beneficial to combat infections. Further studies with a larger number of isolates and various bacterial species may provide a deeper understanding of the molecular mechanisms of the antimicrobial effects of these drugs and their clinical implications in UTIs.

## Supplementary Information

Below is the link to the electronic supplementary material.

Supplementary file1 (DOCX 742 KB)

## Data Availability

No datasets were generated or analysed during the current study.
