# Supplementary material for: Effects of empagliflozin and metformin on biofilm formation and pathogenicity factors of urinary Escherichia coli isolates
Source: Folia Microbiol (Praha). 2026 Jun 3;71(4):847–61. doi: 10.1007/s12223-026-01520-3 (PMC13541807; doi:10.1007/s12223-026-01520-3)
Supplement: Supplementary file 1 [file 12223_2026_1520_MOESM1_ESM.docx]

**Effects of Empagliflozin and Metformin on Biofilm Formation and Pathogenicity Factors of Urinary *Escherichia coli* Isolates**

**Supplement Table 1.** Descriptive information and antibiotic susceptibility profiles of urinary *E. coli* isolates.

| **Isolate No** | **Types of  Clinical Specimens** | **Clinical department/service** | **CFU/mL** | **CXM** | **FT** | **AMP** | **TZP** | **CXMA** | **CRO** | **IMP** | **MEM** | **ERT** | **FOS** | **AK** | **GN** | **CIP** | **SXT** |
| --- | --- | --- | --- | --- | --- | --- | --- | --- | --- | --- | --- | --- | --- | --- | --- | --- | --- |
| **1** | Midstream Urine | Anesthesia Service | 10^4^ | R | S | R | S | R | I | S | S | S | S | S | S | S | R |
| **2** | Midstream Urine | Pediatric Emergency  Service | 10^4^ | R | S | R | S | R | R | S | S | S | S | S | S | R | S |
| **3** | Midstream Urine | Pediatric Emergency  Service | 10^5^ | R | S | R | S | R | R | S | S | S | S | S | R | R | R |
| **4** | Catheter-Urine | Internal Medicine Service | 10^5^ | R | S | R | S | R | I | S | S | S | S | S | S | S | R |
| **5** | Midstream Urine | Emergency Service | 10^6^ | S | S | S | S | S | S | S | S | S | S | S | S | S | S |
| **6** | Midstream Urine | Urology Service | 10^6^ | R | S | R | S | R | I | S | S | S | S | I | S | R | R |
| **7** | Midstream Urine | Internal Medicine Service | 10^6^ | S | S | S | S | S | S | S | S | S | S | S | S | S | S |
| **8** | Midstream Urine | Emergency Service | 10^6^ | S | S | S | S | S | S | S | S | S | S | S | S | S | S |
| **9** | Midstream Urine | Radiation Oncology  Polyclinic | 10^6^ | R | S | R | S | R | I | S | S | S | S | S | S | S | S |
| **10** | Midstream Urine | Urology Service | 10^5^ | S | S | S | S | S | S | S | S | S | S | S | S | S | S |
| **11** | Midstream Urine | Urology Service | 10^4^ | R | S | R | S | R | I | S | S | S | S | S | S | S | R |
| **12** | Midstream Urine | Emergency Service | 10^3^ | R | S | R | S | R | R | S | S | S | S | S | S | S | R |
| **13** | Midstream Urine | Child Health and Diseases Polyclinic | 10^6^ | R | S | R | S | R | I | S | S | S | S | S | S | R | S |
| **14** | Midstream Urine | Pediatric Emergency  Service | 10^4^ | S | S | S | S | S | S | S | S | S | S | S | S | S | S |
| **15** | Midstream Urine | Thoracic Surgery Service | 10^6^ | R | S | R | S | R | R | S | S | S | S | S | R | S | R |
| **16** | Midstream Urine | Urology Polyclinic | 10^6^ | R | S | R | I | R | I | S | S | S | S | S | S | R | R |
| **17** | Midstream Urine | Emergency Service | 10^6^ | S | S | S | S | S | S | S | S | S | S | S | S | S | S |
| **18** | Catheter-Urine | Emergency Service | 10^4^ | R | S | R | S | R | I | S | S | S | S | S | S | S | R |
| **19** | Midstream Urine | Pediatric Surgery Service | 10^6^ | R | S | R | I | R | I | S | S | S | S | S | S | R | R |
| **20** | Catheter-Urine | Pediatric Surgery  Polyclinic | 10^5^ | R | S | R | I | R | R | S | S | S | S | I | R | R | R |
| **21** | Midstream Urine | Pediatric Surgery Service | 10^5^ | R | S | R | R | R | R | S | S | S | R | R | S | S | S |
| **22** | Midstream Urine | Pediatric Surgery Service | 10^5^ | S | S | S | S | S | S | S | S | S | S | S | S | S | S |
| **23** | Midstream Urine | Urology Service | 10^4^ | S | S | S | S | S | S | S | S | S | S | S | S | S | S |
| **24** | Midstream Urine | Obstetrics and Gynecology Polyclinic | 10^5^ | R | S | R | R | R | R | S | S | S | S | S | S | R | S |
| **25** | Midstream Urine | Nephrology Polyclinic | 10^6^ | S | R | S | S | S | S | S | S | S | S | S | S | S | S |
| **26** | Midstream Urine | Child Nephrology  Polyclinic | 10^6^ | S | S | S | S | S | S | S | S | S | S | S | S | S | S |
| **27** | Midstream Urine | Urology Polyclinic | 10^5^ | R | S | R | S | R | R | S | S | S | S | S | S | R | S |
| **28** | Midstream Urine | Child Health and Diseases Polyclinic | 10^5^ | R | S | R | R | R | R | S | S | S | S | S | R | R | S |
| **29** | Midstream Urine | Gastroenterology Service | 10^4^ | S | S | S | S | S | S | S | S | S | S | S | S | S | S |
| **30** | Midstream Urine | Internal Medicine Service | 10^6^ | R | S | R | S | R | I | S | S | S | S | R | S | S | S |
| **31** | Midstream Urine | Pediatric Emergency  Service | 10^6^ | S | S | S | S | S | S | S | S | S | S | S | S | S | S |
| **32** | Midstream Urine | Emergency Service | 10^6^ | R | S | R | S | R | R | S | S | S | S | S | S | I | S |
| **33** | Midstream Urine | Child Health and Diseases Polyclinic | 10^6^ | R | S | R | I | R | R | S | S | S | S | S | R | R | S |
| **34** | Midstream Urine | Obstetrics and Gynecology Polyclinic | 10^6^ | R | S | R | R | R | I | S | S | S | S | S | S | S | S |
| **35** | Midstream Urine | Obstetrics and Gynecology Polyclinic | 10^6^ | S | S | S | S | S | S | S | S | S | S | S | S | S | S |
| **36** | Midstream Urine | Urology Polyclinic | 10^6^ | R | S | R | S | R | R | S | S | S | S | I | S | R | S |
| **37** | Midstream Urine | Emergency Service | 10^6^ | R | S | R | I | R | I | S | S | S | S | I | R | R | S |
| **38** | Midstream Urine | Cardiology Service | 10^6^ | S | S | S | S | S | S | S | S | S | S | S | S | S | S |
| **39** | Midstream Urine | Nephrology Polyclinic | 10^6^ | R | S | R | S | R | R | S | S | S | S | S | S | S | S |
| **40** | Midstream Urine | Child Health and Diseases Polyclinic | 10^6^ | S | S | S | S | S | S | S | S | S | S | S | S | S | S |

*CFU: Colony Forming Unit, AK: Amikacin, AMP: Ampicillin, CIP: Ciprofloxacin, CRO: Ceftriaxone, CXM: Cefuroxime, CXMA: Cefuroxime axetil, ERT: Ertapenem, FOS: Fosfomycin, FT: Nitrofurantoin, GN: Gentamicin, IMP: Imipenem, MEM: Meropenem, SXT: Trimethoprim/Sulfamethoxazole, TZP: Piperacillin/Tazobactam. S: Susceptible, standard dose. I: Susceptible, high dose. R-Resistant.*

**Supplement Table 2.** Effect of MET on *fimH, luxS* and *rpoC* expression at sub-MICs.

| **Isolate** | **MET (MIC/2)** | | | **MET (MIC/4)** | | |
| --- | --- | --- | --- | --- | --- | --- |
|  | *fim*H | *lux*S | *rpo*C | *fim*H | *lux*S | *rpo*C |
| **E15** | 3.0↓ | 2.3↓ | 2.0↓ | 2.1↓ | 2.2↓ | 2.2↓ |
| **E18** | 3.1↓ | 2.4↓ | 1.9↓ | 3.6↓ | 3.7↓ | 2.3↓ |
| **E24** | 3.1↓ | 2.4↓ | 1.9↓ | 4.1↓ | 4.1↓ | 2.2↓ |
| **E34** | 3.4↓ | 3.1↓ | 2.4↓ | 4.2↓ | 3.1↓ | 2.4↓ |
| **E37** | 3.5↓ | 3.7↓ | 2.4↓ | 3.6↓ | 2.2↓ | 2.2↓ |

**Supplement Table 3.** Effect of EMP on *fimH, luxS* and *rpoC* expression at sub-MICs.

| **Isolate** | **EMP (MIC/2)** | | | **EMP (MIC/4)** | | |
| --- | --- | --- | --- | --- | --- | --- |
|  | ***fim*H** | ***lux*S** | ***rpo*C** | ***fim*H** | ***lux*S** | ***rpo*C** |
| **E15** | 1.3↓ | 1.4 ↓ | 1.4↓ | 1.2↓ | 1.3↓ | 1.4↓ |
| **E18** | 1.5↓ | 1.4↓ | 1.3↓ | 1.4↓ | 1.3↓ | 1.4↓ |
| **E24** | 1.4↓ | 1.6↓ | 1.4↓ | 1.2↓ | 1.2↓ | 1.4↓ |
| **E34** | 1.6↓ | 1.9↓ | 1.9↓ | 1.4↓ | 1.4↓ | 1.2↓ |
| **E37** | 3.4↓ | 3.4↓ | 3.3↓ | 1.4↓ | 1.2↓ | 1.6↓ |

**Supplement Table 4.** Primer efficiency parameters for primers.

| **Gene** | **Primer type** | **Slope** | **R²** | **Efficiency (%)** | **Interpretation** |
| --- | --- | --- | --- | --- | --- |
| *fimH* | Target | −3.23 | 0.99 | 103.98 | Acceptable |
| *lux*S | Target | −3.35 | 0.99 | 98.84 | Acceptable |
| *rpo*C | Reference | -3.41 | 0.99 | 96.45 | Acceptable |
| *pol*B | Reference | -3.29 | 0.99 | 101.3 | Acceptable |

**Supplement Table** **5**. Comparison of raw C_T_ values between untreated control and DMSO control groups for *fimH*, *luxS*, and *rpoC* genes.

| **Isolate** | ***fimH***  (Mean C_T_ ±SD) | | ***luxS***  (Mean C_T_ ±SD) | | ***rpoC***  (Mean C_T_ ±SD) | |
| --- | --- | --- | --- | --- | --- | --- |
|  | Untreated Control | DMSO Control | Untreated Control | DMSO Control | Untreated Control | DMSO Control |
| **E15** | 22.10 ± 0.21 | 22.12 ± 0.24 | 24.05 ± 0.35 | 24.08 ± 0.31 | 18.22 ± 0.12 | 18.25 ± 0.14 |
| **E18** | 22.32 ± 0.20 | 22.35 ± 0.18 | 24.38 ± 0.25 | 24.42 ± 0.28 | 18.41 ± 0.19 | 18.44 ± 0.22 |
| **E24** | 22.05 ± 0.30 | 22.08 ± 0.35 | 24.11 ± 0.22 | 24.15 ± 0.19 | 18.29 ± 0.15 | 18.32 ± 0.18 |
| **E34** | 22.40 ± 0.25 | 22.44 ± 0.22 | 24.34 ± 0.30 | 24.38 ± 0.33 | 18.48 ± 0.20 | 18.51 ± 0.25 |
| **E37** | 22.15 ± 0.27 | 22.18 ± 0.29 | 24.19 ± 0.23 | 24.22 ± 0.26 | 18.16 ± 0.10 | 18.19 ± 0.12 |

C_T_: Cycle threshold; SD: standard deviation. The control group contained DMSO at a final concentration (<5% v/v). Statistical analysis (t-test) showed no significant differences (*p* > 0.05) between the untreated control and DMSO control groups for any of the tested genes or isolates,

**Supplement Fig. 1.** Sample collection and selection of *E. coli* isolates for the antibiofilm activity and gene expression analyzes.

**Supplement Fig. 2.** Gel electrophoresis images for *hly, cnf,* and *pap* genes.

**Supplement Fig. 3** ERIC-PCR gel electrophoresis images showing the clonal distribution of forty *E.coli* isolates.

Gel electrophoresis images represent the ERIC-PCR profiles of 40 *E. coli* isolates included in the initial screening. Based on the band patterns, isolates were categorized into 12 unique clones. Lanes marked 'Ladder' indicate the molecular weight marker (100-3000 bp). These raw images support the non-clonal selection of representative isolates (E15, E18, E24, E34, and E37) used in the study.
